# Metastatic undifferentiated pleomorphic sarcoma diagnosed by endoscopic ultrasound‐guided fine‐needle aspiration

**DOI:** 10.1002/jgh3.12818

**Published:** 2022-10-03

**Authors:** Takeru Hirao, Kenji Ikezawa, Ryoji Takada, Tomoyuki Otsuka, Mizuki Korematsu, Shigenori Nagata, Kazuyoshi Ohkawa

**Affiliations:** ^1^ Department of Hepatobiliary and Pancreatic Oncology Osaka International Cancer Institute Osaka Japan; ^2^ Department of Medical Oncology Osaka International Cancer Institute Osaka Japan; ^3^ Department of Head and Neck Surgery Osaka International Cancer Institute Osaka Japan; ^4^ Department of Diagnostic Pathology and Cytology Osaka International Cancer Institute Osaka Japan

**Keywords:** extracolonic lesion, pancreatic metastasis, transcolonic endoscopic ultrasound‐guided fine‐needle aspiration, undifferentiated pleomorphic sarcoma

## Abstract

Pathological differentiation is important for suspected lesions of metastatic undifferentiated pleomorphic sarcoma (UPS) because no reliable imaging criteria exist for this entity yet. In the present case, transgastric endoscopic ultrasound‐guided fine needle aspiration (EUS‐FNA) for the pancreatic tumor and transcolonic EUS‐FNA for the intraabdominal tumor contributed to the definitive diagnosis of metastatic UPS, leading to appropriate treatment selection.

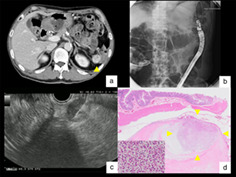

## Introduction

Undifferentiated pleomorphic sarcoma (UPS), previously known as malignant fibrous histiocytoma, used to be the most common malignant soft tissue tumor in adults. It frequently occurs in the superficial soft tissue of the extremities.^1^ Moreover, although surgical resection is the main treatment for UPS, local recurrence after incomplete resection has been reported in 13–52% of the cases, as well as distant metastasis in 31–35% of the cases.^2^, ^3^ The common sites of metastatic UPS are the lung, lymph nodes, bone, and liver, whereas metastasis to intra‐abdominal spaces other than the liver is quite rare (metastasis to the pancreas: 4%).^4^ Furthermore, pathological examinations are essential because no specific imaging criteria for UPS have yet been established.^5^

## Case Report

**Figure 1:**
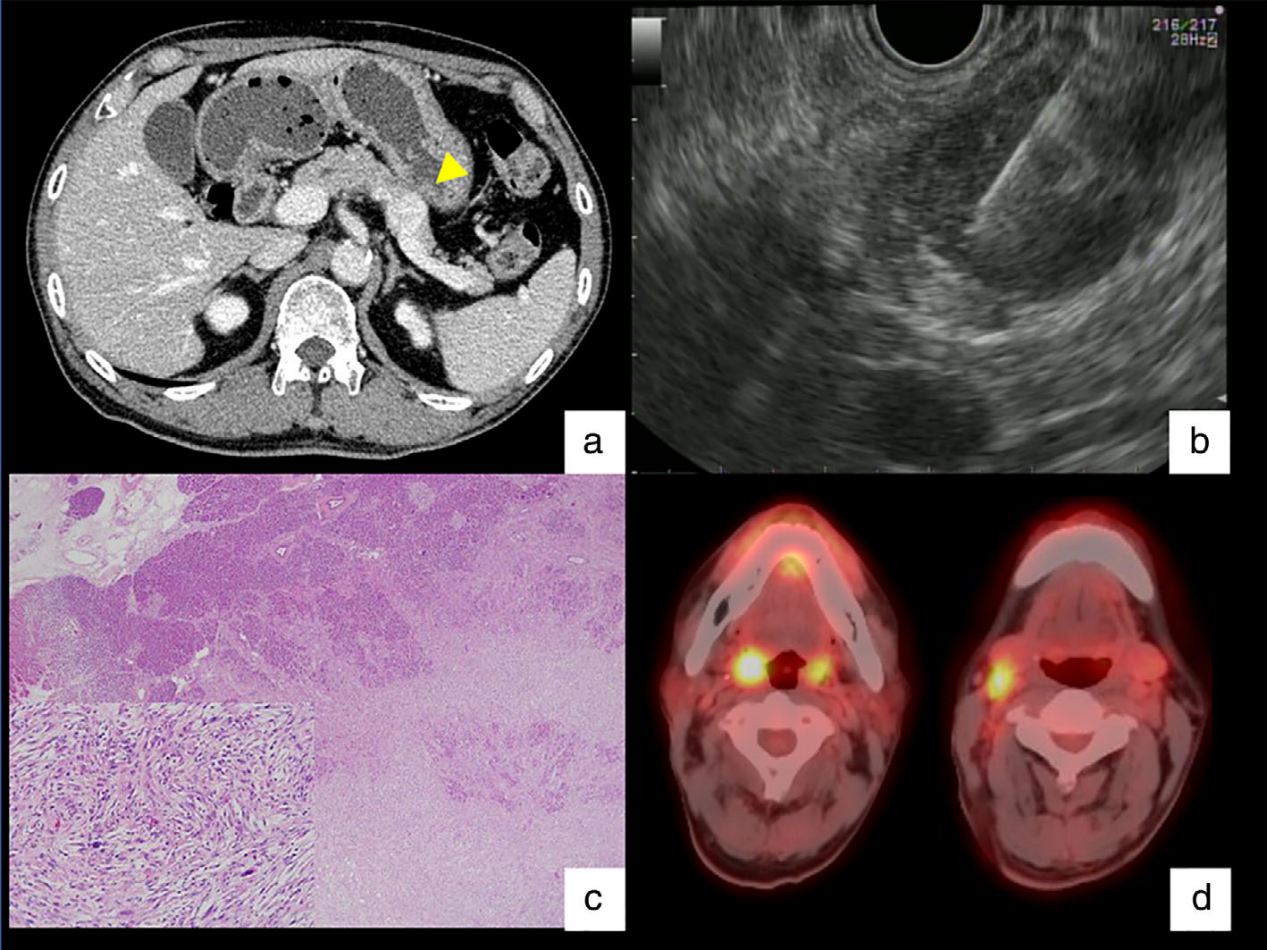
(a) Contrast‐enhanced computed tomography (CT) scan showing a 20‐mm solid mass in the pancreatic tail (arrowhead). (b) Transgastric endoscopic ultrasound‐guided fine needle aspiration for a hypoechoic pancreatic tumor. (c) Photomicrograph of metastatic UPS infiltrating the pancreatic parenchyma (hematoxylin and eosin [HE] stain, ×20), histologically demonstrating spindle‐cell neoplasms with a storiform pattern and focal nuclear pleomorphism (HE, ×200 [small window in the left lower corner]). (d) Positron emission tomography–CT scan showing ^18^F‐fluorodeoxyglucose uptake in the right palatine tonsil and cervical lymph nodes.

**Figure 2:**
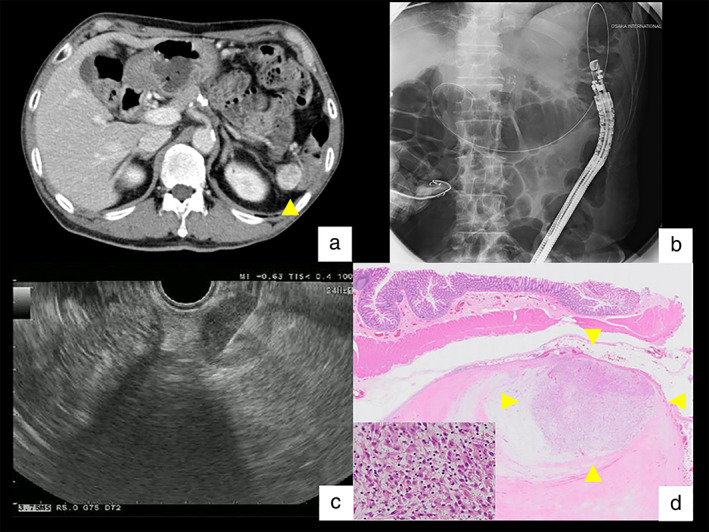
(a) Contrast‐enhanced computed tomography (CT) scan showing a 25‐mm hypervascular tumor located ventral to the left anterior renal fascia (arrowhead). (b) Insertion of convex‐array echoendoscopes (GF‐UCT260; Olympus) to the splenic flexure under fluoroscopic guidance. (c) Transcolonic endoscopic ultrasound‐guided fine‐needle aspiration for a hypoechoic abdominal mass. (d) Photomicrograph of undifferentiated pleomorphic sarcoma (arrowheads) located in the paracolic region (HE, loupe view) with a small window of higher magnification in the lower left corner (HE, ×200), showing proliferation of spindle‐shaped rhabdoid cells admixed with inflammatory infiltrates.

A Japanese man in his 60s underwent resection of a soft‐tissue sarcoma in the left shoulder, which was pathologically diagnosed as UPS. One year and seven months after surgery, contrast‐enhanced computed tomography (CECT) revealed a pancreatic tail mass pathologically diagnosed through endoscopic ultrasound‐guided fine‐needle aspiration (EUS‐FNA) as recurrent UPS metastasizing to the pancreas. The mass was resected by distal pancreatectomy (Fig. 1a–c). Positron emission tomography–computed tomography (PET/CT) scan showed ^18^F‐fluorodeoxyglucose uptake in the right palatine tonsil and cervical lymph nodes (Fig. 1d), leading to a diagnosis of oropharyngeal cancer, which was treated with chemoradiotherapy. One year after treatment, CT revealed multiple pulmonary nodules and mediastinal lymphadenopathy. Through endobronchial ultrasound‐guided transbronchial needle aspiration, these were pathologically diagnosed as a recurrence of the oropharyngeal cancer and were treated with systemic chemotherapy. Eleven months after the initiation of chemotherapy, CECT showed a 25‐mm hypervascular tumor located ventral to the left anterior renal fascia (Fig. 2a). The patient also underwent colonoscopy and transcolonic EUS‐FNA to evaluate tumor involvement in the colorectum. No tumor invasion of the mucosal surface was detected by conventional colonoscopy (PCF‐H290ZI; Olympus Optical Co. Ltd., Tokyo, Japan), which was followed by the insertion of convex‐array echoendoscopes (GF‐UCT260; Olympus) into the splenic flexure under fluoroscopic guidance. Transcolonic EUS‐FNA of the intra‐abdominal tumor revealed a proliferation of spindle‐shaped or flame‐like cells with large nuclei and myxoid matrix, which was diagnosed as high‐grade sarcoma (Fig. 2b,c). No adverse effects of EUS‐FNA were noted. The patient underwent tumor excision along with partial resection of the descending colon, leading to a pathological diagnosis of the second recurrence of UPS (Fig. 2d). Three years after this resection, the patient continued chemotherapy for oropharyngeal cancer without another recurrence of the sarcoma.

## Discussion

Here, transgastric EUS‐FNA for the pancreatic tumor and transcolonic EUS‐FNA for the intra‐abdominal tumor contributed to the definitive diagnosis of metastatic UPS, leading to appropriate treatment selection. Although EUS‐FNA has become a standard method for pancreatobiliary diseases from the upper gastrointestinal tract, yielding pathological details with high diagnostic rates and safety, few reports exist on EUS‐FNA for pancreatic UPS.^6^, ^7^ Transcolonic EUS‐FNA for extracolonic lesions is still challenging due to technical difficulties. Nevertheless, these can be overcome through the combined use of conventional colonoscopy and convex‐array echoendoscope under fluoroscopic guidance.^8^ Recently, Thinrungroj *et al*. reported that forward‐viewing therapeutic linear echoendoscopy‐EUS could be a promising option for easy and safe procedures.^9^ Despite having adverse effects, transcolonic EUS‐FNA for extracolonic lesions could provide important information on differential diagnosis and treatment selection.^10^

Pathological differentiation is important for suspected lesions of metastatic UPS because no reliable imaging criteria exist for this entity yet. Furthermore, EUS‐FNA could be useful for the accurate diagnosis and appropriate management of UPS rarely located in the intra‐abdominal space.
